# Elevated IL-10 is Linked With the Expansion of T-bet^high^CD21^low^ B Cells in Patients With Common Variable Immunodeficiency

**DOI:** 10.1007/s10875-026-02058-2

**Published:** 2026-08-15

**Authors:** Victoria Cousin, Anna Graumann, Valerie Geiger, David Sökler, Patrick Bez, Sylvia Gutenberger, Cornelia Glaser, Geoffroy Andrieux, Melanie Boerries, Björn Christian Frye, Gernot Zissel, Celia Lourdes Calvillo, Javier Rodriguez-Ubreva, Esteban Ballestar, Fabian Hauck, Reinhard E. Voll, Nina Chevalier, Baerbel Keller, Klaus Warnatz

**Affiliations:** 1 Department of Rheumatology and Clinical Immunology, Medical Center - University of Freiburg, Faculty of Medicine, University of Freiburg, Freiburg, Germany; 2 Center for Chronic Immunodeficiency (CCI), Medical Center - University of Freiburg, Faculty of Medicine, University of Freiburg, Freiburg, Germany; 3 Faculty of Biology, University of Freiburg, Schaenzlestrasse 1, 79104 Freiburg, Germany; 4 Spemann Graduate School of Biology and Medicine, University of Freiburg, Freiburg, Germany; 5 Department of Medicine (DIMED), University of Padova, Padua, Italy; 6 Institute of Medical Bioinformatics and Systems Medicine, Medical Center - University of Freiburg, Faculty of Medicine, University of Freiburg, Freiburg, Germany; 7 German Cancer Consortium (DKTK), Partner Site Freiburg, a partnership between, DKFZ and Medical Center - University of Freiburg, Freiburg, Germany; 8 Clinic for Pneumology, Faculty of Medicine, University Medical Center Freiburg, Freiburg, Germany; 9 Epigenetics and Immune Disease Group, Josep Carreras Leukaemia Research Institute (IJC), Badalona, 08916 Barcelona, Spain; 10 Epigenetics in Inflammatory and Metabolic Diseases Laboratory, Health Science Center (HSC), East China Normal University (ECNU), Shanghai, China; 11 Division of Pediatric Immunology and Rheumatology, Department of Pediatrics, Dr. von Hauner Children’s Hospital, University Hospital, Ludwig-Maximilians-Universität (LMU), Munich, Germany

**Keywords:** IL-10, Tbet^high^CD21^low^ B cells, CD21^low^ B cells, Common variable immunodeficiency (CVID), Survival, B-cell differentiation, Type 1 inflammation, Immune dysregulation

## Abstract

Common variable immunodeficiency (CVID) is frequently complicated by autoimmune and inflammatory manifestations associated with profound immune dysregulation (CVIDc), including expansion of T-bet^high^CD21^low^ B cells (CD21^low^ B cells). Elevated serum IL-10 levels have repeatedly been reported in CVIDc, yet their relationship to CD21^low^ B-cell differentiation remains unclear. In this study, we determined a significant correlation between increased serum IL-10 levels and frequency of circulating CD21^low^ B cells, particularly marked in CVIDc patients. Transcriptomic and protein analyses identified multiple cellular sources of IL-10 in CVID, including monocytes, T cells, and a subset of CD21^low^ B cells in peripheral blood and inflamed tissues. CD21^low^ B cells expressed elevated levels of IL-10 receptor subunits and displayed intact IL-10–induced STAT3 signaling, indicating preserved responsiveness to IL-10. Functionally, IL-10 alone neither induced CD21^low^ B-cell differentiation nor inhibited IFN-γ–driven polarization. However, in vitro differentiation assays showed that IL-10 can substitute for IL-21 during CD40L/T cell–dependent differentiation of CD21^low^-like B cells and promote their survival in vitro. These findings suggest that IL-10 may support the expansion and persistence of CD21^low^ B cells at inflammatory sites. Collectively, our data identify IL-10 as a component of the inflammatory environment associated with CD21^low^ B-cell expansion and suggest that IL-10 can functionally replace IL-21 during their differentiation and promote their survival in CVID-associated immune dysregulation.

**Supplementary Information:**

The online version contains supplementary material available at 10.1007/s10875-026-02058-2.

## Introduction

Based on clinical presentation, common variable immunodeficiency (CVID) is classified into patients who present only with infections (CVIDio) and patients with additional autoimmune, lymphoproliferative and inflammatory complications (CVIDc) [1–3]. The latter manifestations are typically associated with specific alterations of the immune homeostasis. Thus, CVIDc patients frequently present with a more severe decrease of switched memory B cells, expansion of T-bet^high^CD21^low^ B cells, reduction of naïve CD4^+^ T cells and a shift towards type 1 immunity [4–7].

One of the serum proteins notably increased in the serum of CVIDc patients is interleukin 10 (IL-10) [8–11]. IL-10 is produced by various cells of the innate and the adaptive immune system, including monocytes, CD4^+^, CD8^+^ T cells, and B cell subsets [12]. The pleiotropic cytokine with both immunosuppressive and stimulatory functions plays a central role in maintaining immune homeostasis. It dampens inflammation by suppressing the production of pro-inflammatory cytokines such as interferon gamma (IFN-γ), IL-2, IL-3, and tumor necrosis factor (TNF-α), inhibits T-cell activation, and preserves intestinal homeostasis [13]. Beyond this regulatory role, IL-10 is a potent stimulator of B cell differentiation, promoting antibody production in the context of T-dependent immune responses partly through autocrine signaling [14, 15]. Elevated IL-10 serum levels have been associated with chronic autoimmune conditions such as systemic lupus erythematosus (SLE) and rheumatoid arthritis (RA) as well as chronic infectious diseases such as HIV [16–18]. Notably, the same chronic disease conditions have also been associated with the expansion of CD21^low^ B cells. Since their description more than 20 years ago in patients with HIV [19] and CVID [20], an expansion of these B cells, partly termed “extrafollicular”, “atypical”, “double negative” [21] or murine “age-associated” B cells [22], have been described in many chronic inflammatory diseases [23]. As shown by our group and others, these cells exhibit a fundamentally altered transcriptional, BCR-signaling and glycosylation profile [24–29] when compared to conventional CD21^pos^ B cells. These cells show a reduced proliferation capacity, residual immunoglobulin production and can serve as potent antigen-presenting cells [30, 31]. We have identified an essential role of IFN-γ-producing T helper cells and B-cell intrinsic T-bet expression for the differentiation of CD21^low^ B cells [5, 26]. CD21^low^ B cells have been found enriched in inflamed tissues such as the bronchioalveolar lavage (BAL) fluid of CVIDc patients, the nephritic kidney in SLE or the synovial fluid of RA patients [30, 32].

Given the concomitant elevation of IL-10 and CD21^low^ B cell counts in different disorders, we aimed to explore the potential relation between increased IL-10 and CD21^low^ B cell differentiation and expansion. Herein, we show here that in CVID the percentage of circulating CD21^low^ B cells correlates with serum IL-10 levels, which is produced by a broad range of immune cells, including a CD21^low^ B cell subset itself. We show that IL-10 can increase the survival of CD21^low^ B cells and replace IL-21 in CD40 mediated differentiation of CD21^low^ B cells and may therefore play a role in their expansion at inflammatory sites with high IL-10 but limiting IL-21 levels.

## Material and Methods

### Patient Cohort and Ethics

Overall, 73 patients (female n = 37, male n = 36; median age 53 ± 16) fulfilling the diagnostic criteria for CVID (www.ESID.org) were included in the study. Patients were classified as CVIDio or CVIDc [3] and as CVID smB-21lo or 21norm according to the EUROclass classification [6]. Control blood was obtained from healthy donors (HD). Detailed clinical characteristics of the study cohort are provided in Supplementary Table S1, including available genetic testing results and the identified variants considered relevant to the patients’ clinical phenotype. In addition to peripheral blood samples, lymph node samples from patients with CVID (CVID LN) and tonsil samples from non-CVID individuals (non-CVID Ton) who underwent tonsillectomy were included as activated tissue controls for comparative analyses. 2 patients diagnosed with sarcoidosis, and 6 patients with RA were included for tissue analysis. All experiments were performed with ethical approval by local authorities 251/13 and 354/19 according to the Declaration of Helsinki. Written informed consent was obtained from all patients and healthy individuals.

### Cytokine Detection

Serum samples were stored at –80 °C. IL-10 was detected by multiplex bead technology assays using the Luminex® xMAP® platform performed by Eve Technologies Corporation, Canada. In a targeted proteomic approach serum level of 172 unique proteins through proximity extension immunoassay (PEA; Olink Proteomics, Sweden), using the panels Olink® Target 96 Immune Response and Immuno oncology were measured. For the detailed description of the technique, please refer to Berbers et al. [10]. This study included 45 individuals, comprising 25 patients with CVID and 10 HD. Patients were classified as CVIDio (n = 10), CVIDc B⁺ CD21norm (n = 7), CVIDc B⁺ CD21lo (n = 12), or CVIDc B⁻ (n = 6). Clinical complications included bronchiectasis (10/25), splenomegaly (20/25), granulomatous disease (4/25), granulomatous-lymphocytic interstitial lung disease (GLILD; 13/25), enteropathy (13/25), chronic norovirus infection (4/25), autoimmune cytopenias (12/25), and other autoimmune manifestations (19/25). Genetic results when available allowed us to identify variants in TNFRSF13B and NFKB1.The results are expressed as normalized protein expression value (NPX) on a log-2 scale.

### Cell Isolation, Culture and Activation

Peripheral Blood Mononuclear Cells (PBMCs) were isolated from EDTA blood by Ficoll density gradient centrifugation following standard protocols. For in vitro cultures, naïve B cells were isolated using the naïve B-Cell Isolation Kit II (Miltenyi Biotec, Germany) following the manufacturer’s instructions (purity > 93%). After isolation, cells were resuspended in Iscove’s Modified Dulbecco’s Medium (IMDM, Life Technologies, United States), 10% fetal calf serum (FCS, Biochrom, Germany), bovine insulin (Sigma-Aldrich, United States), Apo-transferrin (Sigma-Aldrich), non-essential amino acids (Life Technologies), L-glutamine (ThermoFisher, United States), L-glutathione (Sigma-Aldrich) as described [26] and incubated at 7.5% CO_2_. For B cell activation 20.000–30.000 naïve B cells were stimulated with 5 µg/ml anti-IgM (Jackson, United States), 50 ng/ml IFN-γ (Biolegend, United States), trimerized rhCD40L (optimal concentrations, self-made), 50 ng/ml rhIL-21 (Miltenyi, Germany) and 50 ng/ml rhIL-10 (Biolegend) for 48 h. For in vitro differentiation 13.000–16.000 naïve B cells were stimulated with 5 µg/ml anti-IgM, 50 U/ml rhIL-2 (Biolegend), 10 ng/ml BAFF (Biolegend), optimal concentrations of CD40L, 1 µg/ml CpG (Invivogen, United States), 10 ng/ml IL-21, 10 ng/ml IL-10 and 20 ng/ml IFN-γ for 6 days as described previously [26].

### Flow Cytometry

Antibodies used in this study: anti-CD8 BUV496 (RPA-T8), anti-CD27 BV605 (L128), anti-CD27 BUV395 (L128), anti-CD45RA FITC (SH9), anti-CD86 PE (2331 [FUN-1]), anti-CXCR5 AF488 rat (RF8B2), anti-Stat3(pY705) PE (4/P-STAT3) all purchased from Becton, Dickinson and Company (BD Biosciences, United States). Anti-CD3 AF700 (HIT3a), anti-CD3 PerCp/Cy5.5 (SK7), anti-CD3 BV650 (UCHT1), anti-CD4 PE/Cy7 (RPA-T4), anti-CD4 BV785 (RPA-T4), anti-CD8 APC/Cy7 (SK1), anti-CD10 BV605 (HI10a), anti-CD11c AF488 (Bu15), anti-CD19 PE-Cy7 (J4.119), anti-CD19 APC/Cy7 (HIB19), anti-CD19 BV421 (HIB19), anti-CD21 PE/Cy7 (Bu32), anti-CD25 PerCp/Cy5.5 (M-A251), anti-CD25 BV421 (BC96), anti-CD27 BV421 (M-T271), anti-CD38 PerCp/Cy5.5 (HIT2), anti-CD45 BV785 (HI30), anti-CD45RA APC-Cy7 (HI100), anti-CD45RA BV605 (HI100), anti-CD183 (CXCR3) BV421 (G025H7), anti-CD210 (IL-10R) BV421 rat (3F9), anti-CD210b (IL-10RB) PE (S17009F), anti-CD279 (PD-1) APC (EH12.2H7), anti-IL-10 AF647 rat (JES3-9D7), all from Biolegend. Anti-CD27 PE (M-T271), DakoCytomation (Denmark). Anti-CD183 (CXCR3) PE (49801), R&D Systems, United States. Zombie UV (Biolegend); DAPI (4 ‘,6-Diamino-2-phenylindole) (Sigma-Aldrich).

For flow cytometry, cells were stained for 15 min at 4 °C with surface antibodies and washed with PBS 2% FCS. For pSTAT3 signaling experiments, PBMCs were stimulated for 15 min with IL-10 (200 ng/ml). Cytofix and Perm III (BD Biosciences) were used for fixation and permeabilization following the manufacturer’s instructions. Intracellular IL-10 was determined after stimulation of PBMCs with Phorbol 12-myristate 13-acetate (PMA) and Ionomycin in the presence of BFA (all Sigma-Aldrich) for 4 h. Following surface staining, cells were fixed and permeabilized using Cytofix/Cytoperm and Perm/Wash Buffer (BD Biosciences) following the manufacturer’s instructions. Data were acquired using an LSRFortessa (BD Biosciences), and analyzed using FlowJo software (Treestar).

### RNA Sequencing

RNAseq was performed as described previously [26]. In brief, macrophages/inflammatory monocytes, T and B cell subpopulations were sorted from BAL samples of CVID and sarcoidosis patients (Supplementary Fig. S3A). The BAL macrophages/inflammatory monocytes markers expression are shown in Supplementary Fig. S3B. Total RNA was extracted using RNease kits (QIAGEN, Germany) and libraries were prepared with the NEBNext Ultra II Directional RNA Library Prep Kit. Paired-end reads were quality-trimmed using Trimmomatic (v0.38) to remove low-quality bases and adapter sequences [33]. Filtered reads were aligned to the human reference genome (hg38), and gene-level read counts were quantified using STAR (v2.5.2b) [34]. All downstream analyses were conducted in R (v4.2.2). Samples were analyzed separately for each cell type. Genes with zero counts in more than 50% of samples were excluded from further analysis. Differential gene expression analysis was performed using the linear modeling framework implemented in the limma R package [35], with normalization for library size using the trimmed mean of M-values (TMM) method. Gene set enrichment analysis was conducted using the Generally Applicable Gene-set Enrichment (GAGE) R package to identify pathways significantly perturbed between conditions of interest [36]. The full MSigDB repository served as the reference gene set database. These data are now available in the NCBI Gene Expression Omnibus (GEO) under the accession number GSE322936.

Bulk RNA-seq data from peripheral blood naïve CD21^pos^ B cells of HD and patients with CVID, as well as ex vivo CVID CD21^low^ B cells, have been deposited in the GEO under accession number GSE148163. Bulk RNA-seq data from CD8^+^ T cells are available under accession number GSE138076 [37]. Bulk RNA-seq data from TFH cells isolated from secondary lymphoid organs (SLO) of HD and CVID patients have been deposited under accession number GSE274117 [38]. RNA-seq data from duodenal biopsies of HD and CVID patients are available under accession number GSE189820 [39].

Publicly available single-cell RNA sequencing data of PBMCs from CVID patients and healthy donors were obtained, and processed as described in detail in Rodríguez-Ubreva et al. [40]. Samples were analyzed using the Seurat package (v 5.4.0). Average IL-10 gene expression was calculated per cell population and condition (CVID and HD). Cell population percentages were calculated for CVID patients and healthy donors independently. Dotplot used for visualization was created with ggplot2 (v 4.0.1).

### Statistics

For bulk RNAsequencing of BAL samples, differential gene expression analysis was performed using the linear modeling framework implemented in the limma R package [35], with normalization for library size using the trimmed mean of M-values (TMM) method. Normalized log2 cpm for *IL10* were depicted with GraphPad Prism 10 (GraphPad Software Inc.). The statistical annotations shown in the graphs represent adjusted p-values derived from the limma analysis.

Flow cytometry data were analyzed using GraphPad Prism 10. The choice of statistical tests was based on data distribution, assessed using the D’Agostino and Pearson normality test. Comparisons between two groups were performed using the Mann–Whitney U test. For multiple group comparisons, one-way ANOVA followed by Tukey’s post hoc test was applied for normally distributed data, whereas the Kruskal–Wallis test followed by Dunn’s multiple comparisons test was used for non-normally distributed data. The correlation between IL-10 levels and the percentage of CD21^low^ among total B cells was evaluated using simple linear regression.

For the proteomic analysis, we used R version 4.3.1 for the statistics and graphing. Given the non-normal distribution, we compared NPX values of two independent groups by Mann–Whitney U test and for more than two groups Kruskal–Wallis test with post-hoc Dunn test (Dunn.test).

p values of less than 0.05 were considered significant. *p < 0.05, **p < 0.01, ***p < 0.001, and ****p < 0.0001. Error bars in all figures define the means ± SD.

## Results

### IL-10 Levels Correlate with CD21^low^ B Cell Frequency in Peripheral Blood of CVID Patients

**Fig. 1:**
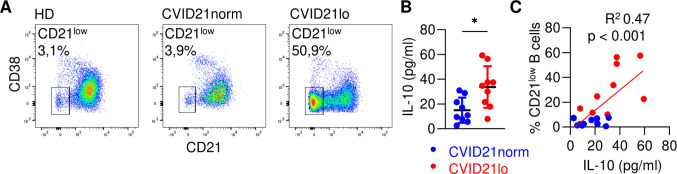
Elevated serum IL-10 levels in CVID patients with expanded CD21^low^ B cells. (**A**) Representative FACS plots of CD21^low^ B-cell population from HD, CVID21norm and CVID21lo patients. (**B**) Serum IL-10 levels CVID21norm (blue), and CVID21lo (red) patients detected by multiplex bead technology. (**C**) Correlation between serum IL-10 levels and the proportion of CD21.^low^ B cells in CVID21norm (blue) and CVID21lo (red). **** p < 0.0001; **p < 0.01

IL-10 was determined in serum from CVID patients with or without an expansion of CD21^low^ B cells (Fig. 1A). These data demonstrated significantly increased IL-10 levels in the serum of CVID21lo patients (> 10% CD21^low^ B cells) compared to CVID21norm patients (< 10% CD21^low^ B cells) (Fig. 1B). The gating strategy is described in Supplementary Fig. S1A. Noticeably, the proportion of CD21^low^ B cells positively correlated with the level of IL-10 suggesting an association of both features (Fig. 1C). With the proteomic approach, we confirmed previous publications showing that IL-10 levels are higher in CVID compared to HDs, and that CVIDc have higher levels than CVIDio patients (Supplementary Fig. S2A). There was no correlation with sex in our cohort (Supplementary Fig. S2A). In particular, CVID patients with any autoimmunity (AI), and more specifically with autoimmune cytopenia (AIC) presented with significantly higher IL-10 levels in the serum compared to patients without AI or AIC, respectively (Supplementary Fig. S2B). Additional complications (AI other than AIC, enteropathy, granuloma formation, granulomatous lymphocytic interstitial lung disease (GLILD), bronchiectasis, or chronic norovirus infection) did not associate with serum IL-10 levels (Supplementary Fig. S2C). The patient cohort is described in Supplementary Table 1. Patients carrying NFKB1 or TNFRSF13B variants did not differ from the remaining CVID patients (Supplementary Fig. S2D).

In summary, increased IL-10 is a common feature of patients with CVID. The levels were higher in CVIDc compared to CVIDio patients, and especially increased in patients with AIC. In particular, IL-10 levels were elevated in CVID21lo versus CVID21norm patients, and correlated with the proportion of CD21^low^ B cells.

### IL-10 Production is Elevated in Various Immune Cell Subsets

**Fig. 2:**
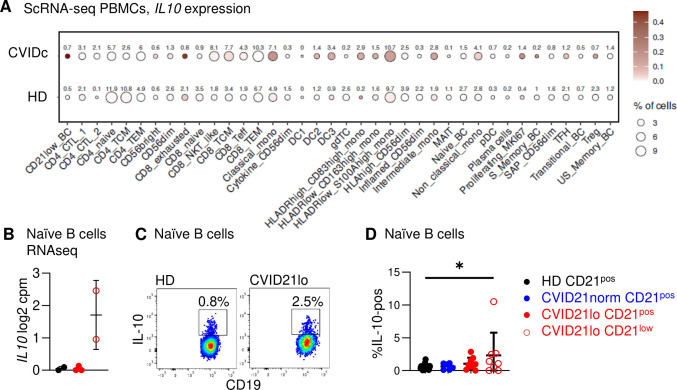
IL-10 expressing cells in peripheral blood. (**A**) *IL10* expression assessed by scRNA sequencing across peripheral blood cell subsets from HD and CVIDc patients. (**B**) *IL10* expression measured by bulk RNA sequencing in HD CD21^pos^ B cells (black), CVID21lo CD21^pos^ cells (red, filled circles), and CVID21lo CD21^low^ B cells (red, open circles). (**C**) Representative FACs plots showing IL-10 expression in naïve B cells from one HD and one CVID21lo patient. (**D**) Percentage of IL-10 positive cells among naïve HD CD21^pos^ B cells (black), CVID21norm CD21^pos^ (blue), CVID21lo CD21^pos^ (red, filled circles), and CVID21lo CD21.^low^ B cells (red, open circles). * p < 0.05

Next, we aimed to explore the source of elevated IL-10 in peripheral blood and inflamed tissue of CVIDc patients. Re-analysing previously published scRNA-seq data [40] from PBMCs of CVIDc patients revealed that increased *IL10* transcripts were detectable in numerous immune cell subsets including monocyte subsets, DCs, Tregs and proliferating cells, with subsets of CD21^low^ B cells and exhausted CD8^+^ T cells expressing the highest levels of *IL10* (Fig. 2A). Consistently, *IL10* transcripts were higher in bulk RNA-seq of naïve CD21^low^ B cells from CVIDc compared to CD21^pos^ from CVIDc and HD (Fig. 2B). Corroborating these transcriptional data, the proportion of IL-10 positive cells after restimulation of PBMCs with PMA and Ionomycin were significantly increased in the CVID naïve CD21^low^ subset compared to HD naïve CD21^pos^ B cells (Fig. 2C and 2D).

**Fig. 3:**
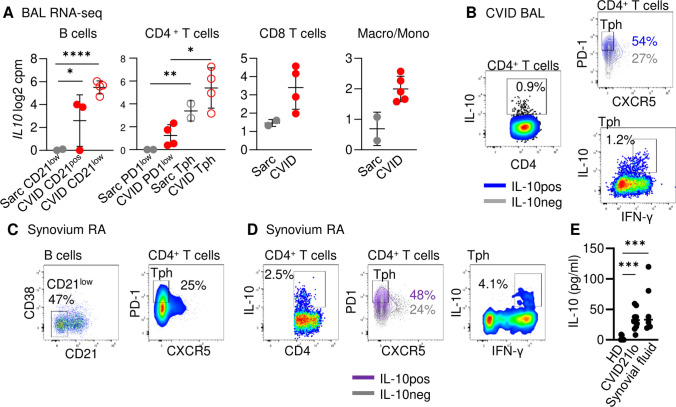
IL-10 expressing cells in inflamed tissues. (**A**) From left to right: *IL10* expression assessed by bulk RNA sequencing in BAL CD21^low^ B cells from sarcoidosis (sarc, gray), CVID21lo CD21^pos^ B cells (red, filled circles), and CVID21lo CD21^low^ B cells (red, open circles); *IL10* expression in BAL PD-1^low^ CD4^+^ T cells from sarcoidosis (gray, filled circles) and CVID (red, filled circles), and in BAL Tph CD4^+^ T cells from sarcoidosis (gray, open circles) and CVID (red, open circles); *IL10* expression in BAL CD8^+^ T cells from sarcoidosis (gray) and CVID (red) patients; *IL10* expression in BAL macrophages/inflammatory monocytes from sarcoidosis (gray) and CVID (red) patients. (**B**) Representative FACS plots showing IL-10–positive CD4^+^ T cells from CVID BAL samples and co-expression of IFN-γ and IL-10 in BAL Tph cells. (**C**) Representative FACS plots of CD21^low^ B cells and CD4.^+^ T cells from RA synovium. (**D**) Representative FACS plots of synovial CD4 T cells and co-expression of IFN-γ and IL-10 in synovial Tph. (**E**) IL-10 serum levels (pg/ml) in HD and CVID 21lo patients, and IL-10 in the synovial fluid of RA patients. **** p < 0.0001, *** p < 0.001, ** p < 0.01, * p < 0.05

Similar to peripheral blood, *IL10* expression was increased across multiple cell populations in BAL fluid from CVIDc patients with interstitial lung disease (Fig. 3A). In the absence of BAL samples from healthy donors, samples from patients with sarcoidosis served as the comparator cohort. BAL-derived CD21^low^ B cells from CVID patients displayed significantly higher *IL10* transcript levels than CD21^low^ B cells from sarcoidosis, while CD21^pos^ B cells from CVID patients showed intermediate expression. CXCR5^neg^PD-1^hi^ peripheral helper T cells (Tph), a population expanded in BAL from CVID patients [41], also expressed high levels of *IL10* compared with Tph cells from sarcoidosis and PD-1^neg^CD4^+^ T cells from both patient groups (Fig. 3A). Increased IL-10 production was confirmed at the protein level in BAL-derived Tph cells (Fig. 3B) and in Tph cells isolated from synovial tissue of patients with RA, where these cells co-expressed IL-10 and IFN-γ (Fig. 3C and 3D). Consistent with this, IL-10 concentrations were elevated in synovial fluid of RA patients and reached levels comparable to those detected in the serum of CVID patients with expanded CD21^low^ B cells (Fig. 3E).

Reanalysis of publicly available bulk RNA-seq datasets from our laboratory further revealed increased *IL10* transcripts in CD8^+^ T cells and a trend toward higher expression in T_FH_ cells from CVID-derived lymph nodes (LN) [38, 42], as well as elevated *IL10* expression in duodenal biopsies from CVID patients [39] (Supplementary Fig. S3B-C).

Together, these findings indicate that IL-10 upregulation in CVIDc extends beyond peripheral blood and is detectable across multiple tissues, with several immune cell subsets (including CD21^low^ B cells) contributing to increased IL-10 production.

CVID B cells express higher levels of IL-10R and respond normally to IL-10.

**Fig. 4:**
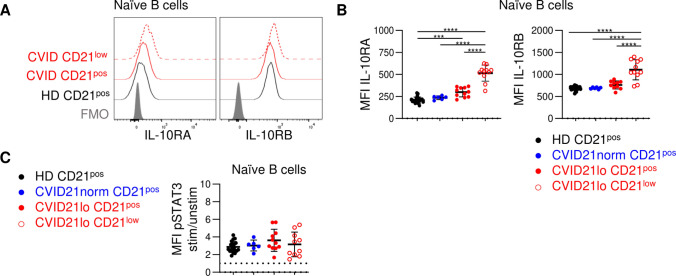
Expression and signaling of IL-10 receptors in naïve B cells. (**A**) IL-10RA and IL-10RB expression in naïve B-cell subset. Comparison between HD CD21^pos^ (black), CVID21lo CD21^pos^ (red, filled line), and CVID21lo CD21^low^ naïve B cells (red, dotted line). (**B**) Mean IL-10 expression in HD CD21^pos^ (black), CVID21norm CD21^pos^ (blue), CVID21lo CD21^pos^ (red, filled circle), and CVID21lo CD21^low^ naïve B cells (red, open circles). (**C**) pSTAT3 signaling in naïve B cells upon IL-10 stimulation. Stimulation index of HD CD21^pos^ (black), CVID21norm CD21^pos^ (blue), CVID21lo CD21^pos^ (red, filled circles), and CVID21lo CD21^low^ (red, open circles) naïve B cells. **** p < 0.0001, *** p < 0.001

Having confirmed an association of CD21^low^ B cell expansion and increased IL-10 levels, we aimed to further explore the capacity of this B cell population to respond to IL-10. Therefore, we first analyzed the expression of IL-10 receptors subunits IL-10RA and IL-10RB, composing the IL-10 receptor, on B cells from HD and CVID patients. In HD, memory B cells express higher levels of both chains than naïve B cells (Supplementary Fig. S4). As most CVID patients lack memory B cells, we compared the expression between different CD27^−^IgD^+^ naïve B cell populations. CD21^low^ B cells from CVID21lo patients displayed significantly higher levels of IL-10RA and IL-10RB compared to naïve CD21^pos^ B cells from HD, CVID21norm and CVID21lo patients (Fig. 4A and 4B). In order to determine the effect of this increased receptor expression on the signaling capacity, we determined the increase of STAT-3 phosphorylation (pSTAT3) after IL-10 stimulation in the different naïve B cell populations. At baseline, naïve CD21^low^ B cells from CVID21lo patients already expressed increased levels of pSTAT3 compared to the other populations (data not shown). After IL-10 stimulation, the stimulation index was comparable between all subsets, implying normal IL-10R signaling (Fig. 4C). Thus, CD21^low^ B cells express higher surface levels of both IL-10 receptor components and are responsive to IL-10 stimulation.

### CD21^low^ B-cell Induction and Survival in the Presence of IL-10

**Fig. 5:**
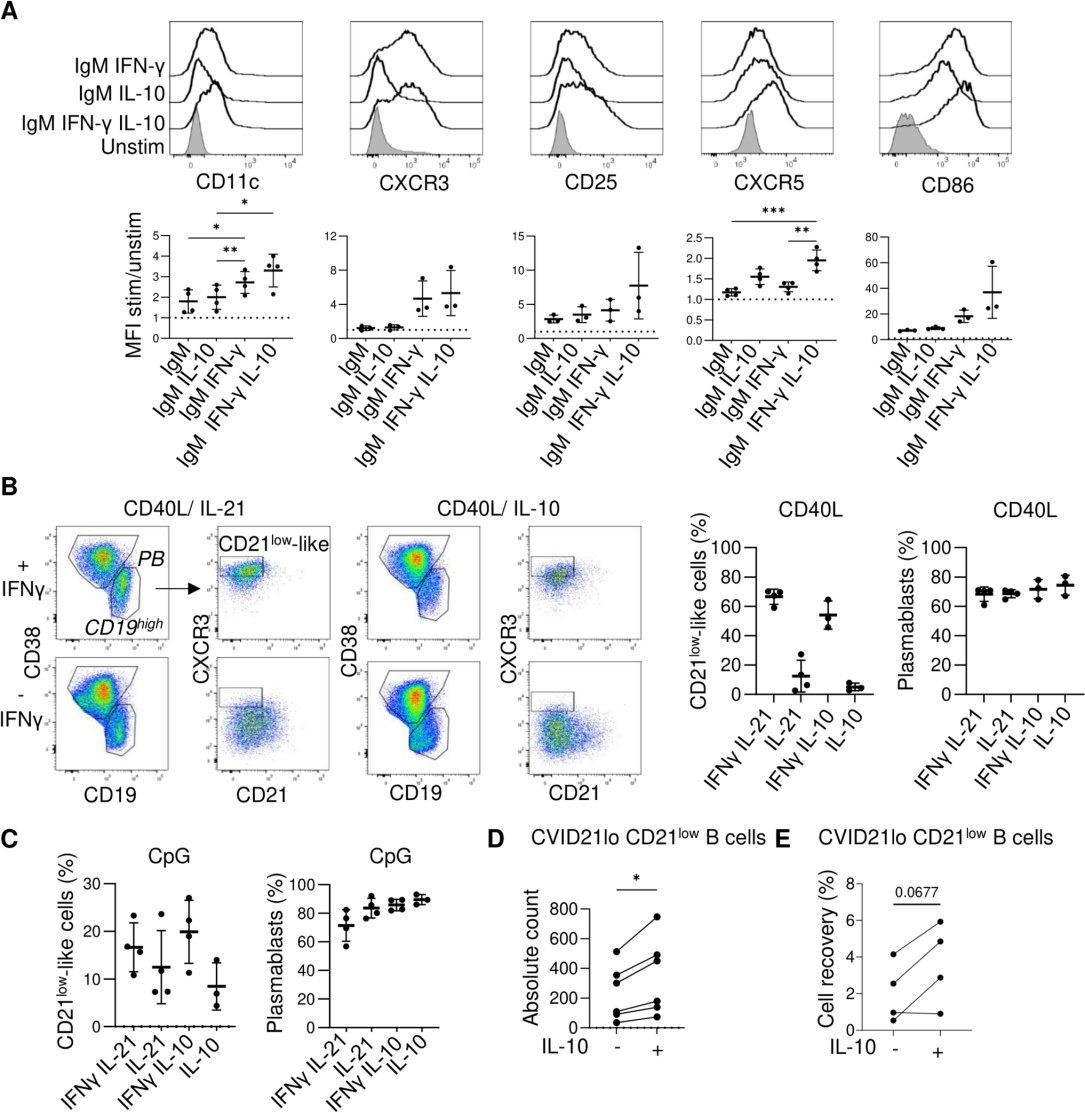
CD21^low^ B-cell induction, differentiation and survival in the presence of IL-10. (**A**) Histogram plots of flowcytometric analysis (top) and stimulation index (bottom) of CD11c, CXCR3, CD25, CXCR5, and CD86 in HD naïve B cells following stimulation with IgM alone, IgM/IL-10, IgM/IFN-γ, or IgM/IFN-γ/IL-10. (**B**) Left: Representative flowcytometric plots showing plasmablasts and CD21^low^-like B cells after 6 days of CD40L-dependent differentiation of HD naïve B cells in the presence of IL-21, IL-10, and/or IFN-γ. Right: Proportions of CD21^low^-like B cells and plasmablasts following differentiation with IFN-γ/IL-21, IFN-γ/IL-10, IL-21 alone, or IL-10 alone. (**C**). Proportions of CD21^low^-like B cells and plasmablasts following 6-day CpG-dependent differentiation after the indicated stimulation. (**D**) Absolute count of CVID21lo CD21^low^ B cells after 2 days of culture in presence of IL-10. (**E**) Cell recovery (%) of CVID21lo CD21.^low^ B cells after 2 days of culture in presence of IL-10. Cell recovery was calculated as the number of final cells divided by the number of initially seeded cells, multiplied by 100. *** p < 0.001; ** p < 0.01; * p < 0.05

As differentiation toward the CD21^low^ phenotype has previously been shown to depend on the synergy between IFN-γ and BCR engagement [26], and given that CD21^low^ B cells are responsive to IL-10, we next investigated whether IL-10 modulates early induction of CD21^low^ B-cell differentiation. Naïve B cells from HD were stimulated with IFN-γ and anti-IgM for two days. IL-10 did not reduce IFN-γ/IgM-induced upregulation of CD11c and CXCR3, indicating that IL-10 does not interfere with the core induction program of CD21^low^ B cells in vitro (Fig. 5A). Consistent with stimulation of HD naïve CD21^pos^ B cells with IL-10 alone (Supplementary Fig. S5A), addition of IL-10 to IFN-γ/IgM stimulation slightly increased CD25 and CD86 expression and significantly enhanced CXCR5 expression (Fig. 5A). IL-10 alone or in combination with IFN-γ/IgM slightly decreased CD27 and IL-10RA expression (Supplementary Fig. S5B).

We next examined whether IL-10 influences full CD21^low^ B-cell differentiation using a six-day culture system in which naïve CD21^pos^ B cells from HD are stimulated with CD40L and IL-21 together with BCR engagement and IFN-γ [26]. In the absence of IFN-γ, neither IL-10 nor IL-21 induced an expansion of CD21^low^-like B cells. However, IL-10 could substitute for IL-21 in promoting differentiation of CD21^low^-like B cells under conditions that promote the generation of CXCR3^high^ CD21^low^-like B cells (Fig. 5B) and similarly supported plasmablast differentiation (Fig. 5B). Following TLR stimulation, IL-10- and IL-21-containing conditions yielded comparable proportions of CD21low-like B cells and plasmablasts (Fig. 5C). Then we investigated whether IL-10 affects survival of CD21^low^ B cells from CVID patients. IL-10 significantly increased the absolute number of CD21^low^ B cells after two days in culture (Fig. 5D). Finally, after 2 days of culture, CVID CD21^low^ B cells showed a trend toward increased cell recovery in the presence of IL-10 (Fig. 5E), indicating improved survival. A representative FACS plot illustrating the Annexin staining strategy used to identify and gate living cells is provided in Supplementary Figure S6.Collectively, these findings indicate that IL-10 does not inhibit T cell–driven differentiation of CD21^low^ B cells and can partially substitute for IL-21 during this process, while also promoting the survival of differentiated CD21^low^ B cells.

## Discussion

This study identifies a previously unrecognized association between elevated serum IL-10 levels and the expansion of CD21^low^ B cells in CVID patients with secondary non-infectious complications and suggests a potential causal relationship between both events. Consistent with previous reports [8, 10, 29, 43–45], we observed elevated serum IL-10 levels in CVID patients compared to HD, with the highest levels observed in CVIDc patients, particularly in those with a history of AIC, as previously reported [46]. In our earlier work [32], we had already reported that AIC in CVID was associated with increased frequencies of CD21^low^ B cells. Stratification according to EUROclass further showed that CVID21lo patients displayed significantly higher serum IL-10 levels, which directly correlated with the proportion of circulating CD21^low^ B cells.

CD21^low^ B cells express high levels of both IL-10 receptor chains and retain intact IL-10 signaling capacity, as indicated by normal STAT3 phosphorylation upon stimulation. Thus, CD21^low^ B cells like other B cells are responsive to IL-10 stimulation. In line with previous work on IL-10 in B-cell activation and differentiation [14, 47, 48], IL-10 alone induced only limited phenotypic changes and did not directly drive differentiation.

IL-10 is generally considered a regulatory cytokine capable of counteracting IFN-γ–driven inflammation [49, 50]. However, its regulatory capacity can diminish once inflammatory responses become chronic. For example, macrophages from chronically infected mice fail to suppress TNF-α and IL-6 production in response to IL-10 despite normal IL-10 receptor expression (51). Consistent with this context-dependent function, IL-10 did not inhibit CD21^low^ B-cell differentiation in our experimental systems. Instead, IL-10 was able to substitute for IL-21 during T-cell–driven differentiation, supporting not only plasma cell differentiation but also the generation of CD21^low^ B cells in vitro. These findings suggest that in the chronically inflamed environment of CVID, IL-10 may not act purely as an anti-inflammatory signal but provide supportive signals for CD40 mediated CD21^low^-B-cell activation and differentiation programs originally initiated by IFN-γ and BCR engagement. In addition, IL-10 alone increased the number of viable CD21^low^ B cells derived from CVID patients in short-term cultures, suggesting improved survival in an IL-10-rich milieu.

This observation is particularly relevant given that increased IL-10 production is detected at several inflammatory sites, including BAL in GLILD, duodenal biopsies from CVID patients with enteropathy, LN of CVID patients with lymphadenopathy, and synovial fluid of RA patients (this manuscript; [37, 39, 52, 53]. Notably, most of these sites (except the gastrointestinal tract) are also characterized by the accumulation of CD21^low^ B cells [30, 41, 54]. These observations suggest that elevated IL-10 and CD21^low^ B-cell expansion frequently co-occur in chronically inflamed tissues.

Le Coz et al. [46] proposed that the elevated plasma IL-10 in CVID originates primarily from circulating CXCR5^hi^PD1^int^CD25^hi^ TFH cells producing both IL-10 and IL-21. Our data extend this concept by identifying multiple cellular sources of IL-10 including monocytes, effector memory CD8^+^ T cells [42] and a subset of CD21^low^ B cells themselves. CD21^low^ B cells have been observed as a source of IL-10 also in the EAE mouse model [55] and in humans with malaria [29]. In BAL, Tph cells appear to represent a relevant source of IL-10, producing both IFN-γ and IL-10, similar to BAL-derived CD4^+^ T cells clones in active pulmonary tuberculosis patients [56]. These cells resemble chronically activated IL-10-secreting Th1 effector cells first described in human blood by Häringer et al. [57].

In systemic autoimmune diseases like SLE, IL-10 levels as well as CD21^low^ B cell frequencies correlate with disease activity and autoantibody production [58–60]. In RA, CD21^low^ B cells accumulate in synovial fluid where IL-10 concentrations are increased [61, 62]. While both findings have independently been attributed to chronic type-1 inflammatory environments present both systemically and at inflamed tissue sites [5, 26, 28, 29], our data suggest that IL-10 itself may directly contribute to the differentiation and accumulation of CD21^low^ B cells across diverse immune disorders and that CD21^low^ B cells may directly contribute to elevated IL-10 levels at the site.

Recent studies highlight substantial heterogeneity among T-bet^high^CD21^low^ B cells [23]. Notably, a CD25^pos^T-bet^high^CD21^low^ B cell subset with regulatory properties and IL-10 production has recently been described in mice [55]. Together with our observation that IL-10 stimulation increases CD25 expression under Th1-polarizing conditions, this raises the possibility that IL-10 co-stimulation may additionally influence the differentiation of specific CD21^low^ B-cell subsets in chronic inflammatory environments.

One limitation of this study is the inherent clinical and genetic heterogeneity of patients with CVID. To facilitate interpretation, we provide the genetic and clinical characteristics of all included patients, included only a small number of monogenic cases, and performed the functional in vitro experiments exclusively in patients with complex CVID (CVIDc) to reduce biological heterogeneity. While such heterogeneity may contribute to differences in the cellular sources and systemic effects of IL-10, it is less likely to influence the cell-intrinsic molecular mechanisms underlying the effects of IL-10 on CD21^low^ B-cell biology. In addition, our in vitro experiments, especially in regard to the survival of CD21^low^ B cells, cannot fully recapitulate the complexity of the inflamed tissue microenvironment, where multiple cytokines, stromal cells, and immune cell interactions collectively shape B-cell function. The relatively limited number of tissue samples and the use of disease controls for selected analyses should also be considered when interpreting the findings. In conclusion, we demonstrate a correlation between serum IL-10 levels and the frequency of circulating T-bet^high^CD21^low^ B cells in CVID patients. Multiple immune cell populations (including a subset of CD21^low^ B cells themselves) contribute to elevated IL-10 levels in this type-1 inflammatory disease. Rather than inhibiting CD21^low^ B-cell differentiation, IL-10 can support their expansion by partially substituting for IL-21 during differentiation, and by enhancing survival of differentiated CD21^low^ B cells, suggesting that IL-10 may act as a context-dependent signal that promotes accumulation of these cells within chronically inflamed tissues.

## Supplementary Information

Below is the link to the electronic supplementary material.

Supplementary file1 (PDF 1.64 MB) **Figure S1. **Gating strategy for peripheral T and B cells. (A) Representative FACS plots from a healthy donor (HD; upper panel) and a CVID21lo patient (lower panel), depicting T- and B-cell subpopulations.

Supplementary file2 (PDF 1.21 MB) **Figure S2. **IL-10 serum levels. (A) Left: Serum IL-10 levels in HD (black), CVIDio (gray) and CVIDc patients (pink). Color code is used in all subsequent parts of this figure; Right: Comparison of serum IL-10 levels in male and female CVID patients. (B) Serum IL-10 levels in CVID patients with autoimmunity (AI) and with or without autoimmune cytopenia (AIC). (C) Serum IL-10 levels in CVID patients with or without one of the following conditions: AI other than AIC, enteropathy, granuloma, GLILD, bronchiectasis, norovirus complications. (D) Serum IL-10 levels in CVIDio or CVIDc patients with TNFRSF13B or NFKB1 variants. **** p < 0.0001; *** p < 0.001; ** p < 0.01; * p < 0.05

Supplementary file3 (PDF 3.14 MB) **Figure S3.** IL10 transcripts are elevated in inflamed tissues, including BAL, lymph nodes and duodenal biopsies of CVID patients. (A) Gating strategy used to sort BAL derived CD21low and CD21pos B cells, CD8+ T cells, macrophages/ inflammatory monocytes, Tph and CXCR5 PD1low CD4+ T cells for bulkRNA sequencing. (B) Marker gene expression of sorted BAL derived macrophage/monocyte populations for bulk RNA sequencing. (C) IL10 transcripts in TFH cells and CD8+ T cells from non-CVID tonsils (non-CVID Ton, black) and CVID lymph nodes (LN, red). (D) IL10 transcripts from duodenal biopsies of HD (black) and CVID patients with enteropathy (red). *** p < 0.001; * p < 0.05

Supplementary file4 (PDF 107 KB) **Figure S4. **Expression of IL-10RA and IL-10RB in HD B cell subpopulations. (A) Mean fluorescence expression of IL-10RA and IL-10RB in naïve, IgM memory, Switched memory and double-negative (DN) subpopulations. **** p < 0.0001, *** p < 0.001

Supplementary file5 (PDF 396 KB) **Figure S5. **Effect of IL-10 on HD CD21pos B cells. (A) Mean expression of activation markers CD86, CXCR5, and CD25 on HD CD21pos naïve B cells after 48 h stimulation with IL-10. Each color represents an individual donor. (B) Expression of HLA-DR, IL-10RA and CD27 after two-day stimulation with IgM alone, IgM/IL-10, IgM/IFN-γ, or IgM/IFN-γ/IL-10. ** p < 0.01; * p < 0.05

Supplementary file6 (PDF 347 KB) **Figure S6.** Representative FACs plot showing living, apoptotic and dead B cells after 2 days in culture with IL-10. (A) FACS plots from a healthy donor (HD; left panel) and a CVID21lo patient (right panel)

Supplementary file7 (DOCX 32 KB)

## Data Availability

Bulk RNA-seq data from BAL samples are now available in the NCBI Gene Expression Omnibus (GEO) under the accession number GSE322936. Bulk RNA-seq data from peripheral blood naïve CD21pos B cells of HD and patients with CVID, as well as ex vivo CVID CD21low B cells, have been deposited in the GEO under accession number GSE148163. Bulk RNA-seq data from CD8 + T cells are available under accession number GSE138076. Bulk RNA-seq data from TFH cells isolated from secondary lymphoid organs (SLO) of HD and CVID patients have been deposited under accession number GSE274117. RNA-seq data from duodenal biopsies of HD and CVID patients are available under accession number GSE189820.
