# Supplementary material for: Elevated IL-10 is Linked With the Expansion of T-bethighCD21low B Cells in Patients With Common Variable Immunodeficiency
Source: J Clin Immunol. 2026 Aug 15;46(1):83. doi: 10.1007/s10875-026-02058-2 (PMC13477453; doi:10.1007/s10875-026-02058-2)
Supplement: Supplementary file 2 [file 10875_2026_2058_MOESM2_ESM.pdf]

Supplementary Figure S2

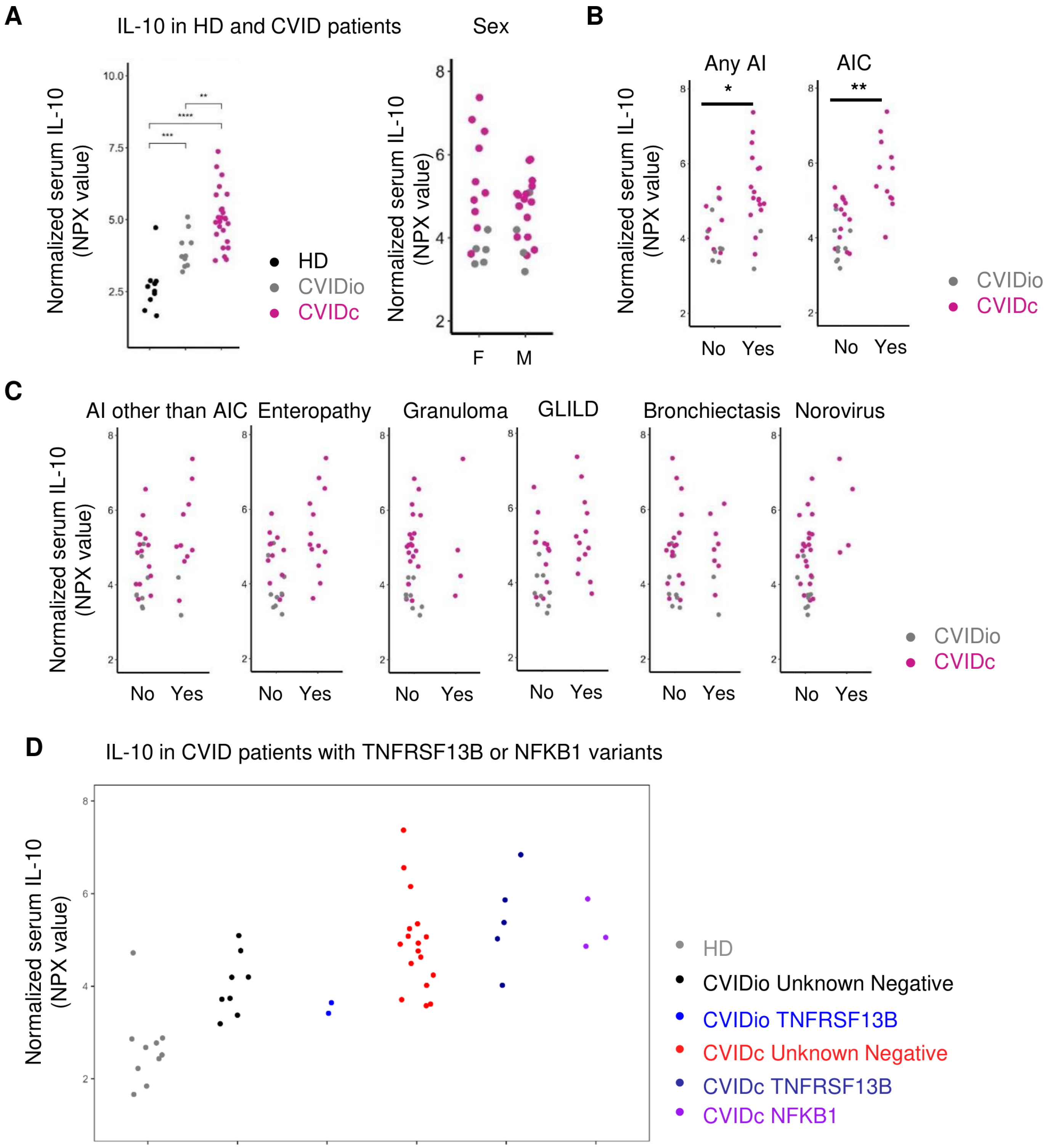

Figure S2. IL-10 serum levels. (A) Left: Serum IL-10 levels in HD (black), CVIDio (gray) and CVIDc patients (pink). Color code is used in all subsequent parts of this figure; Right: Comparison of serum IL-10 levels in male and female CVID patients. (B) Serum IL-10 levels in CVID patients with autoimmunity (AI) and with or without autoimmune cytopenia (AIC). (C) Serum IL-10 levels in CVID patients with or without one of the following conditions: AI other than AIC, enteropathy, granuloma, GLILD, bronchiectasis, norovirus complications. (D) Serum IL-10 levels in CVIDio or CVIDc patients with TNFRSF13B or NFKB1 variants. **** $p < 0.0001$; *** $p < 0.001$; ** $p < 0.01$; * $p < 0.05$
