# Supplementary material for: Elevated IL-10 is Linked With the Expansion of T-bethighCD21low B Cells in Patients With Common Variable Immunodeficiency
Source: J Clin Immunol. 2026 Aug 15;46(1):83. doi: 10.1007/s10875-026-02058-2 (PMC13477453; doi:10.1007/s10875-026-02058-2)
Supplement: Supplementary file 3 [file 10875_2026_2058_MOESM3_ESM.pdf]

Supplementary Figure S3

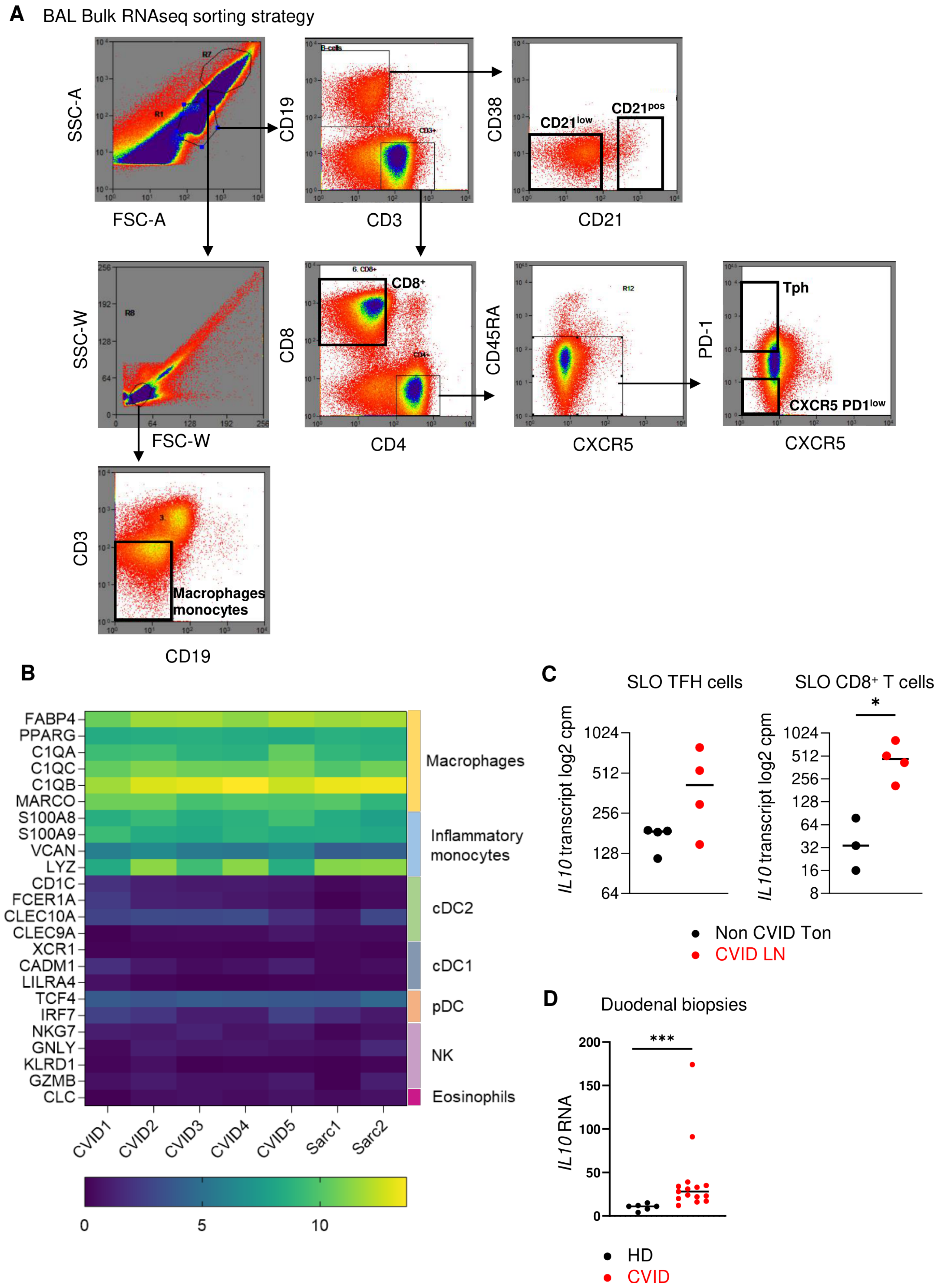

Figure S3. IL10 transcripts are elevated in inflamed tissues, including BAL, lymph nodes and duodenal biopsies of CVID patients. (A) Gating strategy used to sort BAL derived CD21low and CD21pos B cells, CD8+ T cells, macrophages/ inflammatory monocytes, Tph and CXCR5 PD1low CD4+ T cells for bulkRNA sequencing. (B) Marker gene expression of sorted BAL derived macrophage/monocyte populations for bulk RNA sequencing. (C) IL10 transcripts in TFH cells and CD8+ T cells from non-CVID tonsils (non-CVID Ton, black) and CVID lymph nodes (LN, red). (D) IL10 transcripts from duodenal biopsies of HD (black) and CVID patients with enteropathy (red). *** $p < 0.001$; * $p < 0.05$
