# Supplementary material for: Elevated IL-10 is Linked With the Expansion of T-bethighCD21low B Cells in Patients With Common Variable Immunodeficiency
Source: J Clin Immunol. 2026 Aug 15;46(1):83. doi: 10.1007/s10875-026-02058-2 (PMC13477453; doi:10.1007/s10875-026-02058-2)
Supplement: Supplementary file 4 [file 10875_2026_2058_MOESM4_ESM.pdf]

Supplementary Figure S4

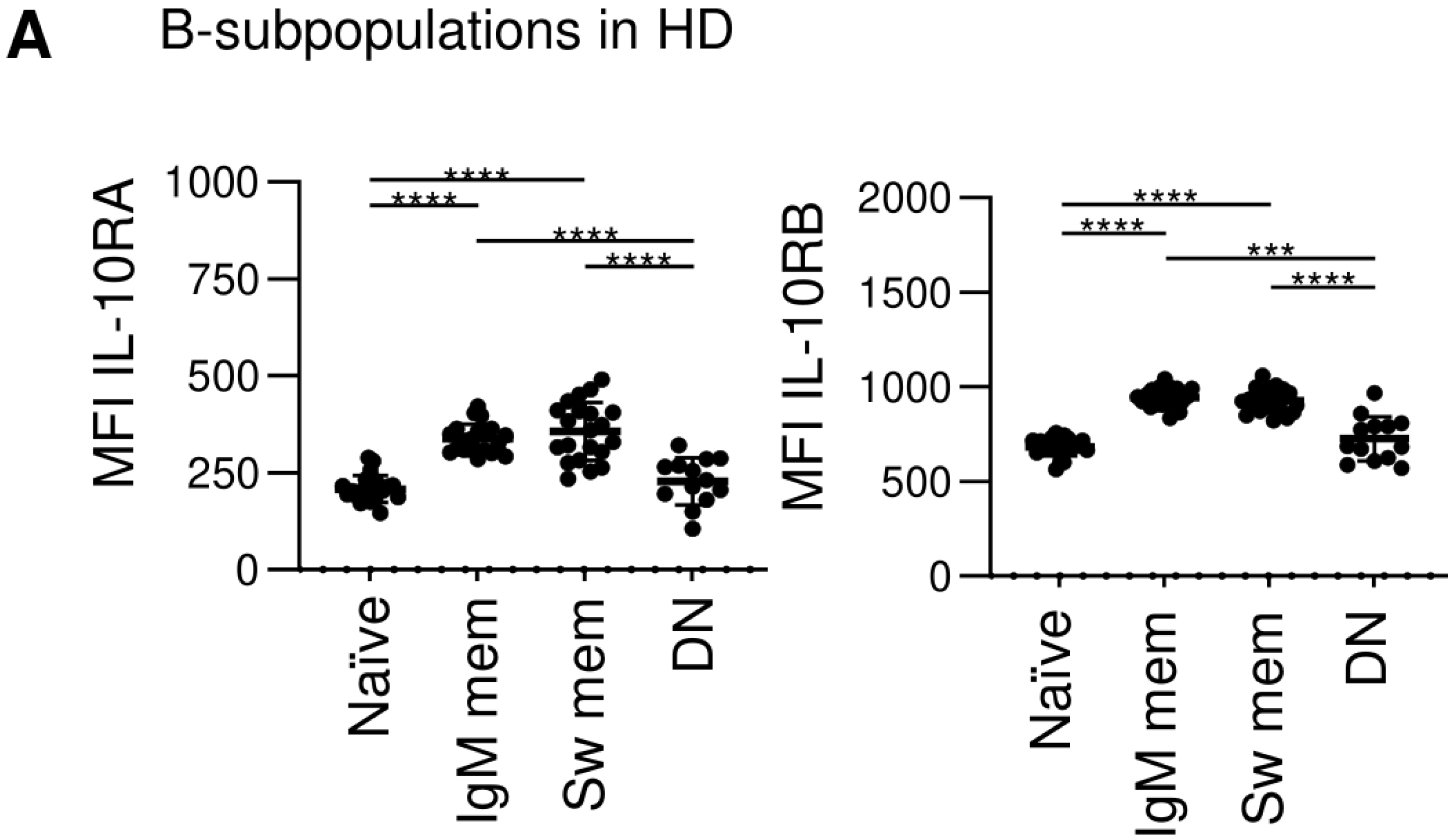

Figure S4. Expression of IL-10RA and IL-10RB in HD B cell subpopulations. (A) Mean fluorescence expression of IL-10RA and IL-10RB in naïve, IgM memory, Switched memory and double-negative (DN) subpopulations. **** $p < 0.0001$, *** $p < 0.001$
