# Supplementary material for: Elevated IL-10 is Linked With the Expansion of T-bethighCD21low B Cells in Patients With Common Variable Immunodeficiency
Source: J Clin Immunol. 2026 Aug 15;46(1):83. doi: 10.1007/s10875-026-02058-2 (PMC13477453; doi:10.1007/s10875-026-02058-2)
Supplement: Supplementary file 5 [file 10875_2026_2058_MOESM5_ESM.pdf]

Supplementary Figure S5

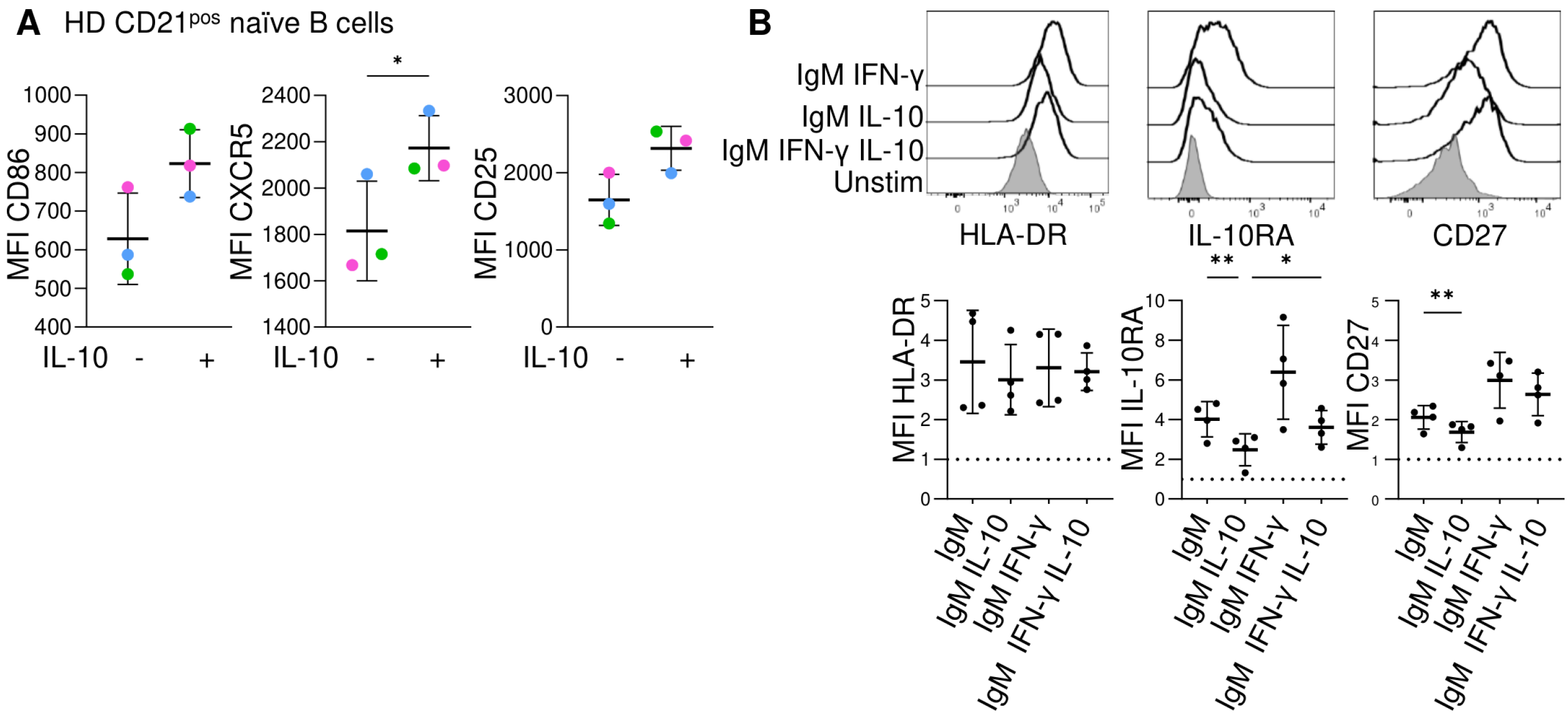

Figure S5. Effect of IL-10 on HD CD21pos B cells. (A) Mean expression of activation markers CD86, CXCR5, and CD25 on HD CD21pos naïve B cells after 48 h stimulation with IL-10. Each color represents an individual donor. (B) Expression of HLA-DR, IL-10RA and CD27 after two-day stimulation with IgM alone, IgM/IL-10, IgM/IFN-γ, or IgM/IFN-γ/IL-10. ** $p < 0.01$; * $p < 0.05$
