# Supplementary material for: Elevated IL-10 is Linked With the Expansion of T-bethighCD21low B Cells in Patients With Common Variable Immunodeficiency
Source: J Clin Immunol. 2026 Aug 15;46(1):83. doi: 10.1007/s10875-026-02058-2 (PMC13477453; doi:10.1007/s10875-026-02058-2)
Supplement: Supplementary file 6 [file 10875_2026_2058_MOESM6_ESM.pdf]

Supplementary Figure S6

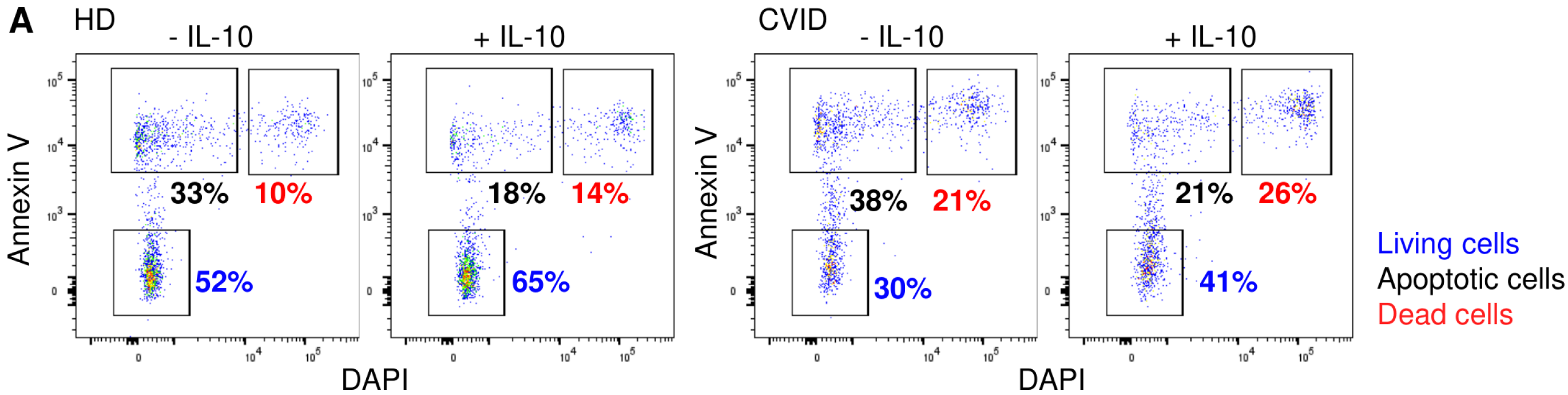

Figure S6. Representative FACs plot showing living, apoptotic and dead B cells after 2 days in culture with IL-10. (A) FACS plots from a healthy donor (HD; left panel) and a CVID21lo patient (right panel)
