# Supplementary material for: Elevated IL-10 is Linked With the Expansion of T-bethighCD21low B Cells in Patients With Common Variable Immunodeficiency
Source: J Clin Immunol. 2026 Aug 15;46(1):83. doi: 10.1007/s10875-026-02058-2 (PMC13477453; doi:10.1007/s10875-026-02058-2)
Supplement: Supplementary file 7 [file 10875_2026_2058_MOESM7_ESM.docx]

| **Sample ID** | **Sex** | **Age** | **Study** | **Clinical classification** | **EUROclass** | **Genetic results** | **Complications** |
| --- | --- | --- | --- | --- | --- | --- | --- |
| **CVID1** | Male | 71 | Proteomics cohort 1 | CVIDc | B- | Negative | Bronchiectasis and secondary pulmonary fibrosis |
| **CVID2** | Male | 62 | Proteomics cohort 1 | CVIDc | B+smB+ 21norm | Negative | Granulomatous disease, GLILD, Splenomegaly, lymphadenopathy, Bronchiectasis |
| **CVID3** | Male | 54 | Proteomics cohort 1 | CVIDc | B+smB- 21low | TNFRSF13B | GLILD, AIC, Splenomegaly, Lymphadenopathy, Lymphoma |
| **CVID4** | Male | 53 | Proteomics cohort 1 | CVIDio | B+smB-21low | Negative | Splenomegaly |
| **CVID5** | Female | 51 | Proteomics cohort 1 | CVIDc | B- | Unknown | GLILD, AIC, Enteropathy, Arthritis, Bronchiectasis |
| **CVID6** | Female | 64 | Proteomics cohort 1 | CVIDc | B+smB-21norm | TNFRSF13B | GLILD, Enteropathy, Arthritis |
| **CVID7** | Female | 53 | Proteomics cohort 1 | CVIDio | B+smB+21norm | TNFRSF13B | Splenomegaly |
| **CVID8** | Male | 47 | Proteomics cohort 1 | CVIDc | B+smB-21norm | Unknown | None |
| **CVID9** | Female | 67 | Proteomics cohort 1 | CVIDio | B+smB+21norm | TNFRSF13B | None |
| **CVID10** | Male | 59 | Proteomics cohort 1 | CVIDc | B+smB-21low | NFKB1 | AIC, Splenomegaly, Arthritis, Bronchiectasis, Vitiligo |
| **CVID11** | Female | 42 | Proteomics cohort 1 | CVIDio | B+smB+21norm | Negative | Bronchiectasis, Type 1 diabetes mellitus |
| **CVID12** | Male | 52 | Proteomics cohort 1 | CVIDc | B+smB-21nlow | NFKB1 | AIC, Splenomegaly, Enteropathy, Hepatopathy, Vitiligo, HPVdriven Anal Ca |
| **CVID13** | Female | 53 | Proteomics cohort 1 | CVIDc | B- | Negative | GLILD, Splenomegaly, Arthritis, Bronchiectasis |
| **CVID14** | Female | 61 | Proteomics cohort 1 | CVIDc | B+smB-21low | TNFRSF13B | Enteropathy, Gastric Ca, Bronchiectasis |
| **CVID15** | Female | 52 | Proteomics cohort 1 | CVIDc | B+smB- 21norm | Negative | Splenomegaly |
| **CVID16** | Male | 56 | Proteomics cohort 1 | CVIDio | B- smB+ 21norm | Negative | None |
| **CVID17** | Male | 49 | Proteomics cohort 1 | CVIDc | B+smB- 21low | Negative | GLILD, Splenomegaly, Enteropathy, Hepatopathy, atrophic gastritic, Gastric Ca, pernicious anemia, Bronchiectasis, Vitiligo |
| **CVID18** | Female | 61 | Proteomics cohort 1 | CVIDc | B+ smB+ 21norm | Negative | AIC, Splenomegaly, Bronchiectasis |
| **CVID19** | Male | 50 | Proteomics cohort 1 | CVIDc | B+smB+21norm | TNFRSF13B | GLILD, AIC, Splenomegaly |
| **CVID20** | Female | 67 | Proteomics cohort 1 | CVIDc | B+smB-21norm | Negative | GLILD, AIC |
| **CVID21** | Male | 54 | Proteomics cohort 1 | CVIDc | B+smB-21norm | TNFRSF13B | GLILD, Enteropathy, Hepatopathy, AIC, Splenomegaly, Bronchiectasis, |
| **CVID22** | Male | 47 | Proteomics cohort 1 | CVIDio | B+smB- 21norm | Negative | None |
| **CVID23** | Female | 42 | Proteomics cohort 1 | CVIDc | B- | Negative | Enteropathy, AIC, Splenomegaly |
| **CVID24** | Female | 66 | Proteomics cohort 1 | CVIDc | B- | Negative | Granuloma, GLILD, Arthritis, Enteropathy, AIC, Splenomegaly |
| **CVID25** | Male | 58 | Proteomics cohort 1 | CVIDc | B+smB- 21low | Unknown | GLILD, splenomegaly, Type 1 diabetes mellitus, cholangiocellular Ca, nno small cell lung Ca |
| **CVID26** | Male | 46 | Proteomics cohort 1 | CVIDio | B+smB-21norm | Negative | None |
| **CVID27** | Male | 71 | Proteomics cohort 1 | CVIDc | B+smB- 21norm | NFKB1 | Enteropathy, Hepatopathy, Splenomegaly |
| **CVID28** | Male | 59 | Proteomics cohort 1 | CVIDc | B+smB-21norm | TNFRSF13B | Granulomatous disease, splenomegaly, Enteropathy |
| **CVID29** | Female | 69 | Proteomics cohort 1 | CVIDc | B+smB- 21low | Negative | Granulomatous disease, GLILD, AIC, splenomegaly |
| **CVID30** | Male | 42 | Proteomics cohort 1 Naive B bulkRNA seq | CVIDc | B+ smB- 21low | Negative | GLILD, AIC, lymphoma |
| **CVID31** | Female | 53 | Proteomics cohort 1 | CVIDio | B+ smB+ 21 Norm | Negative | None |
| **CVID32** | Female | 58 | Proteomics cohort 1 | CVIDio | B+ smB- 21norm | Negative | None |
| **CVID33** | Male | 72 | Proteomics cohort 1 | CVIDio | B+smB- 21low | Negative | Psoriasis, Bronchiectasis |
| **CVID34** | Male | 45 | Proteomics cohort 1 | CVIDc | B+smB+ 21low | Negative | GLILD, splenomegaly |
| **CVID35** | Female | 52 | Proteomics cohort 2 | CVIDc | B+smB- 21low | Negative | Granulomatous disease, GLILD, Myelitis, enteropathy, hepatopathy, Splenomegaly |
| **CVID36** | Female | 59 | Proteomics cohort 2 Naive B bulkRNA seq Funtional in vitro assays | CVIDc | B+smB- 21low | Negative | GLILD, Enteropathy, Hepatopathy, AIC |
| **CVID37** | Male | 59 | Proteomics cohort 2 | CVIDc | B+smB- 21low | Negative | Granulomatous disease, AIC, splenomegaly |
| **CVID38** | Female | 49 | Proteomics cohort 2 | CVIDc | B+smB- 21low | TNFRSF13B | Enteropathy, Lymphoma, Encephalitis, Thyroiditis |
| **CVID39** | Male | 50 | Proteomics cohort 2 | CVIDc | B+smB- 21norm | Negative | Enteropathy, Hepatopathy, Splenomegaly |
| **CVID40** | Female | 31 | Proteomics cohort 2 Functional in vitro assays | CVIDc | B+smB- 21low | Negative | Splenomegaly, Lymphadenopathy, GLILD |
| **CVID41** | Female | 42 | Proteomics cohort 2 | CVIDc | B+smB+ 21low | Negative | AIC, Psoriasis, Bronchiectasis |
| **CVID42** | Male | 49 | Proteomics cohort 2 | CVIDc | B+smB- 21low | Negative | GLILD, AIC |
| **CVID43** | Female | 48 | Proteomics cohort 2 | CVIDc | B+smB- 21low | Negative | GLILD, Bronchiectasis, Hepatopathy |
| **CVID44** | Female | 43 | Proteomics cohort 2 | CVIDc | B+smB+2 21norm | Negative | Autoimmune hepatitis, Hepatopathy, AIC, Enteropathy |
| **CVID45** | Male | 63 | Proteomics cohort 2 | CVIDc | B+smB- 21norm | Negative | Neoplasia, Gastritis, Splenomegaly |
| **CVID46** | Female | 37 | Proteomics cohort 2 | CVIDc | B+smB- 21norm | Negative | GLILD, Hepatopathy, Splenomegaly |
| **CVID47** | Male | 41 | Proteomics cohort 2 Functional in vitro assays | CVIDio | B+smB+ 21norm | Negative | Bronchiectasis |
| **CVID48** | Female | 35 | Proteomics cohort 2 | CVIDio | B+smB- 21norm | Negative | Splenomegaly |
| **CVID49** | Male | 63 | Proteomics cohort 2 | CVIDc | B+smB- 21norm | Negative | Atrophic Gastritis, Vitiligo |
| **CVID50** | Female | 45 | Proteomics cohort 2 | CVIDc | B+smB- 21norm | Negative | AIC |
| **CVID51** | Female | 40 | Proteomics cohort 2 | CVIDio | B+smB+ 21norm | Negative | None |
| **CVID52** | Male | 35 | Proteomics cohort 2 | CVIDio | B+smB- 21norm | Negative | Chronic diarrea |
| **CVID53** | Male | 53 | Functional in vitro assays | CVIDio | B+smB- 21low | Negative | Bronchiectasis |
| **CVID54** | Female | 36 | Functional in vitro assays | CVIDio | B+smB-21norm | Negative | None |
| **CVID55** | Male | 48 | Functional in vitro assays | CVIDc | B+smB-21low | TNFRSF13B | Bronchiectasis, Enteropathy, |
| **CVID56** | Female | 35 | Functional in vitro assays BAL RNAseq | CVIDc | B+smB- 21low | Negative | GLILD, Hepatopathy, AIC |
| **CVID57** | Female | 37 | Functional in vitro assays | CVIDio | B+smB+ 21low | Negative | None |
| **CVID58** | Male | 63 | Functional in vitro assays  Proteomics cohort 1 | CVIDc | B+smB+ 21norm | Negative | Chronic diarrhea |
| **CVID59** | Male | 21 | Functional in vitro assays | CVIDc | B+smB- 21low | Negative | Lymphadenopathy, splenomegaly |
| **CVID60** | Female | 25 | Functional in vitro assays | CVIDc | B+smB- 21low | Negative | GLILD, Bronchiectasis, Enteropathy |
| **CVID61** | Female | 72 | Functional in vitro assays | CVIDio | B+smB- 21norm | Negative | None |
| **CVID62** | Male | 34 | Functional in vitro assays | CVIDio | B+smB+ 21norm | Negative | None |
| **CVID63** | Male | 73 | BAL RNAseq | CVIDc | B+smB- 21low | NFKB1 | Chronic diarhea, Splenomegaly, rosacea |
| **CVID64** | Female | 53 | BAL RNAseq | CVIDc | B+smB-21norm | Negative | Bronchiectasis, Obstructive disease, |
| **CVID65** | Female | 56 | BAL RNAseq | CVIDc | B+smB-21low | Negative | Granulomatous disease, GLILD, Hepatopathy,spelnomegaly, Bronchiectasis, Neoplasia |
| **CVID66** | Female | 60 | BAL RNAseq | CVIDc | B+smB-21low | Unknown | Granuloma, GLILD, Hepatopathy |
| **CVID67** | Male | 34 | Naive B RNAseq | CVIDc | B+smB-21low | Negative | GLILD, Hepatopathy, AIC |
| **CVID68** | Male | 54 | Functional in vitro assays | CVIDc | B+smB-21low | Negative | Bronchiectasis, AIC, Splenomegaly |
| **CVID69** | Female | 58 | Functional in vitro assays | CVIDc | B+smB-21low | Negative | Splenomegaly, Lymphadenopathy, AIC, |
| **CVID70** | Female | 44 | Functional in vitro assays | CVIDc | B+smB-21low | Negative | Splenomegaly, ILD |
| **CVID71** | Male | 60 | Functional in vitro assays | CVIDc | B+smB+ 21low | Negative | GLILD, Hepatopathy, AIC |
| **CVID72** | Female | 45 | Functional in vitro assays | CVIDc | B+smB-21low | Negative | GLILD, AIC |
| **CVID73** | Male | 64 | Functional in vitro assays | CVIDc | B+smB-21low | Negative | GLILD, Atrophic gastritis |

**Supplementary Table S1.** Clinical and immunological characteristics of the CVID patient cohort. The table summarizes patient sex, age at sample collection, study cohort, clinical classification (CVIDc or CVIDio), EUROclass immunophenotypic classification, and available genetic results.
